# Practical Medicine

**Published:** 1880-05

**Authors:** 


### Summary.

*Collaborators:*

DR. L. W. CASE, DR. R. TILLEY.

### PRACTICAL MEDICINE.

LECTURE ON EPILEPSY.—By W. R. Gowers, M.D., F.R.C.P.

The third lecture was devoted to pathology and treatment. Pathological anatomy in idiopathic epilepsy gives us little help. Regarding convulsions, however, pathological anatomy and experiment give us two sets of facts regarding the seat of the "discharge." Experiment shows that convulsions may originate in the medulla, from a center near the respiratory. On the other hand, no local diseases cause convulsions so frequently as those of the cortex; and irritation of the cortical motor "centers" may cause convulsions, having definite modes of onset, like those sometimes seen in idiopathic disease. But other experimental facts make it possible that these convulsions, though excitable from the surface, arise in connected lower centers. Hence the latest writer of a systematic account of the disease, Rothnagel, with full knowledge of all pathological and experimental facts, concludes that epilepsy is a disease of the medulla oblongata. But the clinical study of modes of onset shows that at least many attacks commence in the hemispheres, viz., those of which the earliest symptom is a special sense or physical aura. The clinical evidence that attacks ever commence in the medulla is small. The "pneumogastric aura" might be regarded as such evidence, but it may be present in attacks which certainly originate in the hemispheres. Such visceral sensations may accompany physical auræ, as they do normal physical states (*e. g.*, fear). A curious case, however, was narrated, in which fits could be produced by passive movement of the spine, evidence of a probable origin in the medulla. The wide extent of "warnings" makes it probable that, as Dr. Hughlings Jackson has suggested, any of the gray matter of the encephalon which subserves sensori-motor processes may be the seat of the primary discharge in idiopathic epilepsy, but the evidence that the process of the fit commences in the hemispheres is much stronger and more frequent than that it commences lower down.

To explain loss of consciousness, vaso-motor spasm has been assumed, but the hypothesis is unnecessary, since the affection of consciousness may be the result of the discharge. Other evidence that vaso-motor spasm causes any of the symptoms is inconclusive. Pallor of face is not invariable, and does not necessarily show anæmia of brain. It may result from the cerebral discharge. To explain all the symptoms of attacks it is unnecessary to go farther than the instability of the gray matter in which the discharge begins.

This instability may be better understood as instability of resistance than as any increase in the energy-producing function of cells. The doctrine of internal resistance to action has underlain many current expressions as "nerve-tension," and its recognition gives us clearer views of many physiological and pathological problems. Thus we can understand why loss of blood causes convulsions, or a peripheral impression can arrest a fit, not for the moment, but for a long time.

The treatment of epilepsy, it was remarked, is a subject on which numerical analysis gives little help, because so many patients whose fits cease under treatment relapse when treatment is relinquished. The time available permitted little more than a statement of the remedies most useful in 562 cases in which the effect of treatment was carefully noted. The results showed that while we must not rely *exclusively* on bromides, on these our chief trust must still be placed. Of the three alkaline salts the bromide of potassium deserves, as it has received, the first place. The salt of ammonium is more useful, only in proportion to the slightly greater quantity of bromine which it contains, while a careful comparison in a series of cases, between the salts of sodium and potassium showed that the former is distinctly less useful. The maximum effect of each dose of bromide occurs the sooner, the smaller the dose; hence small doses used to be given frequently. Bromide lessens reflex action, perhaps by increasing resistance in the center, since it antagonises strychnia, which is believed to lessen resistance. If the view that unstable resistance is the chief element in epilepsy is correct, increase of resistance may be the explanation of the action of bromide. The mode of administration is usually by continuous course, in doses just sufficient to arrest the fits. Given thus it was rarely found well to give more than a drachm and a half daily; if this does not suffice, combinations with other drugs answered better than increasing the regular dose. But it was urged that a course of large doses may be for a time employed, with a view of obtaining the full nutritive change in the nervous system, which bromide can effect. For this purpose it was thought best to give it in considerable doses, at longer intervals, beginning with half an ounce. The largest single doses given had been of one ounce, the doses being given at intervals of three to five days.

The value of the various combinations of bromide with other drugs had been tested by ascertaining first, during several months, the effect of a given dose of bromide, and then adding to it the agent to be tested. Of the combinations in common use, those with digitalis and belladonna deserve, as they have commonly received, the first place. Digitalis has enjoyed repute in epilepsy for two hundred years. Alone it sometimes does marked good, and in 63 cases the combination of digitalis and bromide was distinctly more useful than bromide only; in 37 of the cases the attacks ceased during the treatment. The effect of this combination is not confined to the cases in which there is cardiac disturbance, although in these it is almost always useful. Digitalis markedly increases the effect of bromide in nocturnal epilepsy. A case was mentioned in which a patient had not had a single fit for two years on the combination, although he had a fit at night every few weeks on bromide only. Belladonna alone will sometimes arrest attacks. The combination of it with bromide was distinctly more useful than bromide alone in 35 cases, and in 15 arrest of the fits was obtained. Indian hemp is now and then of marked service even alone. A case was mentioned in which the attacks always ceased on this drug, and recurred at once when bromide was substituted. Other combinations which had been found useful were bromide with aconite, and bromide with hydrocyanic acid. The cases in which the addition of iodide to bromide increases its effect are rare.

Zinc deserves some of the repute it has enjoyed for more than a hundred years. Of a large number of cases, in which the oxide was used in doses as large as the patient could bear it, it was distinctly useful in ten, but in only three did the attacks cease. Bromide of zinc, in doses which could be borne, and the bromide of camphor, had seemed of small value. The addition of arsenic to bromide in no case had any influence on the fits, although largely used, on account of the certainty with which the bromide rash could thus be lessened or removed. Turpentine (recommended by Dr. Radcliffe) had been found distinctly useful only in hystero-epilepsy. The use of iron in epilepsy was discountenanced by high authorities. In some cases it seemed to increase the attacks, but in the majority in which it had been given its influence was distinctly beneficial. In four cases the attacks ceased on iron only; in eight others iron alone was distinctly better than bromide alone; and in nineteen the addition of iron to bromide had a marked influence; in eleven the attacks, which had persisted on bromide, ceased when iron was added, and remained absent as long as the combination was given. In several inveterate cases, in which bromide had little effect, the lecturer had given borax in doses of ten to fifteen grains twice or three times a day. In some it was useless, in some its beneficial effect was most distinct. Several cases were narrated in which the attacks ceased for a long time under its use. Occasionally it causes gastro-intestinal disturbance, but many patients bear it well. Cocculus indicus had not been tried sufficiently for an opinion to be formed, but very little benefit had been observed from its alkaloid, picrotoxine. The same conclusion was drawn from an interesting series of cases in which Dr. Ramskill had employed it hypodermically, which showed, however, that a dose of eighteen milligrammes will almost invariably produce a fit in from twenty to thirty minutes. Other drugs which had been tried and found useless were benzoate of soda and nitro-glycerine.

In hystero-epilepsy, bromides, sometimes useful, often fail. Belladonna, iron, valerianate of zinc, and turpentine had appeared of greatest value. The treatment of the actual attacks of hystero-epilepsy is often a matter of difficulty. In the slight fits Dr. Pare's plan of closing the mouth and nose is often useful. When this fails Faradization of the skin and cold douches on the head and water poured into the open mouth are often efficacious. Chloroform is of little value. Where all fail the lecturer had found the hypodermic injection of apomorphia invariably successful. After a twelfth of a grain had been injected, in four minutes all spasm was over, in six minutes the patient would get up, in eight minutes vomit, and then, except for slight nausea, be well.

In conclusion, the lecturer remarked that although the condition of many epileptics was still gloomy enough, yet the present generation had witnessed an advance in the treatment of the disease equalled perhaps in no other branch of therapeutics. Thanks to the influence of the drug the use of which in epilepsy was wholly due to fellows of that college, hundreds of sufferers had been cured, and thousands were leading useful lives who would otherwise have been incapacitated by the disease. For all the victims of the disease we might surely trust that the progress of the recent past is the dawn of a brighter day.

